# Genetics of Adaptation of the Ascomycetous Fungus *Podospora anserina* to Submerged Cultivation

**DOI:** 10.1093/gbe/evz194

**Published:** 2019-09-14

**Authors:** Olga A Kudryavtseva, Ksenia R Safina, Olga A Vakhrusheva, Maria D Logacheva, Aleksey A Penin, Tatiana V Neretina, Viktoria N Moskalenko, Elena S Glagoleva, Georgii A Bazykin, Alexey S Kondrashov

**Affiliations:** 1 Department of Mycology and Phycology, Faculty of Biology, Lomonosov Moscow State University, Moscow, Russia; 2 Skolkovo Institute of Science and Technology, Moscow, Russia; 3 Institute for Information Transmission Problems of the Russian Academy of Sciences, Moscow, Russia; 4 Belozersky Institute of Physico-Chemical Biology, Lomonosov Moscow State University, Moscow, Russia; 5 White Sea Biological Station, Lomonosov Moscow State University, Republic of Karelia, Russia; 6 Zoological Museum, Lomonosov Moscow State University, Moscow, Russia; 7 Department of Plant Physiology, Faculty of Biology, Lomonosov Moscow State University, Moscow, Russia; 8 Department of Ecology and Evolutionary Biology, University of Michigan, Ann Arbor

**Keywords:** experimental evolution, *Podospora anserina*, submerged cultivation, adaptation, positive selection

## Abstract

*Podospora anserina* is a model ascomycetous fungus which shows pronounced phenotypic senescence when grown on solid medium but possesses unlimited lifespan under submerged cultivation. In order to study the genetic aspects of adaptation of *P. anserina* to submerged cultivation, we initiated a long-term evolution experiment. In the course of the first 4 years of the experiment, 125 single-nucleotide substitutions and 23 short indels were fixed in eight independently evolving populations. Six proteins that affect fungal growth and development evolved in more than one population; in particular, in the G-protein alpha subunit FadA, new alleles fixed in seven out of eight experimental populations, and these fixations affected just four amino acid sites, which is an unprecedented level of parallelism in experimental evolution. Parallel evolution at the level of genes and pathways, an excess of nonsense and missense substitutions, and an elevated conservation of proteins and their sites where the changes occurred suggest that many of the observed fixations were adaptive and driven by positive selection.

## Introduction

Over the last several decades, experimental evolution became an essential part of evolutionary biology, providing an opportunity to observe and study changes that occur in controlled populations in the course of many generations. In particular, the *Escherichiacoli* long-term evolution experiment led by Richard Lenski since 1988 yielded a number of important results, including that on morphological evolution of cells (Lenski and Travisano 1994; Philippe et al. 2009), long-term fitness gain under a constant environment (Lenski and Travisano 1994; Lenski et al. 2015), and ecological specialization (Blount et al. 2012; Leiby and Marx 2014). The existence of 12 replicas made it possible to study changes in genotypes and phenotypes that occurred in parallel in independently evolving bacterial populations (Lenski and Travisano 1994; Woods et al. 2006; Cooper et al. 2008).

Being easily cultured and fast-growing, *E. coli* serves as a convenient model organism for experimental evolution. Still, the range of questions that can be studied on it and on other prokaryotes is limited by their relative simplicity. Eukaryotes such as protozoans or fungi possess more complex gene regulation, morphology and physiology and have also been used in studies of experimental evolution (Rundle et al. 2005; Dettman et al. 2008; Gray and Cutter 2014; McDonald et al. 2016).

Another convenient taxon for experimental evolution is ascomycetous fungi. Here, evolutionary experiments were conducted on species of various complexity, including unicellular yeasts as well as complex multicellular *Neurospora*. Experimental evolution in this group was used to study different aspects of fungal biology including germination strategies in a changing environment (Graham et al. 2014), mechanisms of protection of multicellular cooperation (Bastiaans et al. 2016), the advantage of parasexual recombination (Schoustra et al. 2007), and the cost of drug resistance (Schoustra et al. 2006). The vast majority of works on experimental evolution in fungi is conducted on solid media. However, fungi behave differently when cultured in different conditions, and the changes accompanying the transition to a new environment are understudied.

Under submerged cultivation, most ascomycetes cease to reproduce either sexually or asexually, instead transitioning to vegetative proliferation. Depending on specific conditions of cultivation, mycelium either grows dispersed or forms spherical structures called pellets (Gibbs et al. 2000). One study performed submerged cultivation of filamentous fungus *Trichoderma citrinoviride* that was selected for efficient conidia formation and dispersed mycelium (Lin et al. 2017), but generally transition to submerged cultivation remains unexplored.

*Podospora anserina* is a model ascomycete fungus that possesses a definite lifespan with pronounced phenotypic senescence when grown on solid medium (Lorin et al. 2006). In contrast, under conditions of submerged cultivation, *P. anserina* displays unlimited lifespan with no signs of senescence (Turker and Cummings 1987; Kudryavtseva et al. 2011). Transition to unlimited lifespan proceeds via three steps. First, upon transition to submerged cultivation, unadapted culture forms spherical corticated pellets pigmented dark due to the synthesis of melanin. Next, pellets become smaller and start losing their dark pigmentation. Finally, the mycelium in the adapted culture becomes light-colored and homogeneous, it partially forms fluffy pellets of different sizes and partially remains diffuse and unstructured. Adapted cultures can be maintained via submerged cultivation indefinitely.

Isolates taken from adapted cultures revert to senescence, suggesting that the unlimited lifespan is at least partially caused by the environment directly or driven by epigenetic changes. Still, such isolates demonstrate increased lifespan when plated on solid medium and become sterile, only rarely forming mostly nonfunctional microconidia and protoperithecia (Turker and Cummings 1987; Kudryavtseva et al. 2011), suggesting that genetic changes may be involved. After a period of submerged cultivation, *P. anserina* cultures acquire higher rates of biomass accumulation and change their pigmentation, and these changes may also be genetic. Still, the mechanisms that alter *P. anserina* senescence program and affect its phenotype under such conditions are unknown.

In order to study the genetic aspects of adaptation that takes place during the submerged cultivation of *P. anserina*, we started in 2012 a long-term evolutionary experiment. We used two wild strains of *P. anserina* to establish eight independent experimental populations. Here, we analyze the genetic changes that took place in the first 4 years of this experiment.

## Materials and Methods

### Evolutionary Experiment

**Fig. 1.**
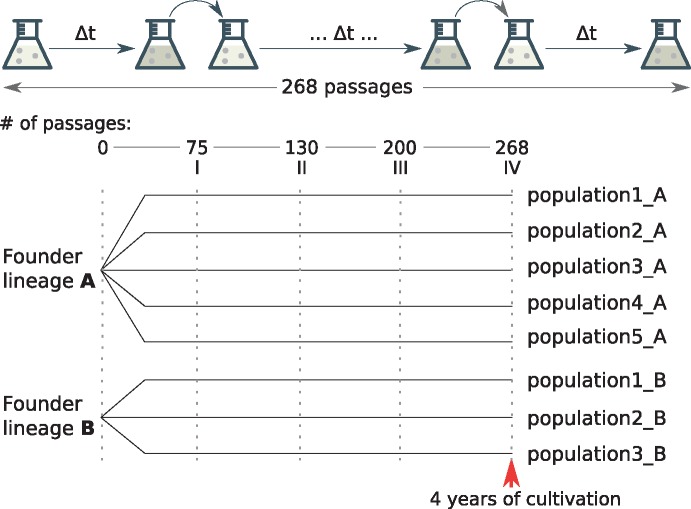
—Scheme of the experiment. Eight experimental populations were established from two founder lineages A and B. After 75, 130, 200, and 268 passages, we determined genotypes of the experimental populations (dashed lines; see Materials and Methods).

Two homokaryotic vegetatively incompatible wild strains *s* and *S* (denoted here as A and B) were kindly provided by Annie Sainsard-Chanet and Carole H. Sellem (Département Biologie Cellulaire et Intégrative, Centre de Génétique Moléculaire, CNRS, Gif-sur-Yvette Cedex, France) and gave rise to five and three independent experimental populations, respectively (fig. 1). Experimental populations were maintained via serial passages and were grown in the dark on the standard synthetic medium M2 (Kudryavtseva et al. 2011). Submerged cultivation was carried out in 750 ml Erlenmeyer flasks with 100 ml of the medium at 27 ± 1°C on a 200 rpm rotor shaker. Cultures were transferred to fresh medium every 4–7 days. During the experiment, mycelium samples were plated on agarized (20 g/l) M2 medium in Petri dishes. Initial founder strains are stored on ararized medium.

We denoted 64, 130, 200, and 268 passages as the first, the second, the third, and the fourth time points of the experiment, respectively. Whole-genome sequencing was performed for both the founder populations, for populations B at the points 1, 2, and 4, and for populations A only at the point 4. The fixations that were observed from whole-genome sequencing data were subsequently verified by Sanger sequencing (see below). Several samples of passage 64 were lost due to contamination; for that reason, we substituted passage 64 with the closest available passage 75 and refer to passage 75 as the first time point in the Results section. The scheme of the experiment is shown in figure 1.

### Whole-Genome Sequencing

DNA was extracted using CTAB-based method (Doyle and Doyle 1987). For library preparation for the first series of samples (founder genotype B and experimental populations B at the first time point), we used Truseq DNA sample preparation kit (Illumina), for the second series of samples (experimental populations B at the second time point) we used NEBNext Ultra DNA kit (New England Biolabs) according to manufacturer instructions. For the third series (founder genotype A and all eight experimental populations at the fourth time point), we constructed PCRfree libraries using Accel-NGS 2S PCR-Free DNA Library Kit (Swift Biosciences). The first and second series were sequenced on HiSeq2000 (Illumina) using Truseq v.3 reagents. The third series was sequenced using Nextseq500 and v.2 reagents.

### De Novo Genome Assembly

Pair-end reads from HiSeq (100 bp) and NextSeq (150 bp) were trimmed using Trimmomatic (Bolger et al. 2014) with options (ILLUMINACLIP: adapters: 2:30:10 LEADING:7 TRAILING:7 SLIDINGWINDOW:5:10 MINLEN:50). Duplicate reads were excluded using fastq-mcf (with -D 50 option).

We performed de novo genome assembly of both founder genotypes with SPAdes (Bankevich et al. 2012) (with –only-assembler option). The initial assemblies were then filtered using the published reference genome (http://podospora.i2bc.paris-saclay.fr, v. 6.31, last accessed ∼ Dec 2014). We used BlastN (Altschul et al. 1990) to find the best hits for each contig in published genome and kept only those contigs that had a local alignment of at least 5 kb with published genome with identity of at least 95%. Finally, we performed correction of assemblies using ICORN2 (Otto et al. 2010). Statistics on assemblies is provided in supplementary table S1, Supplementary Material online.

### Variant Calling

For each of the sequenced samples, we mapped its pair-end reads onto the corresponding founder genome using bwa (Li and Durbin 2009) and removed reads that had nonunique alignments. Average coverage for each sample is provided in supplementary table S1, Supplementary Material online. Filtered mappings were used for variant calling. We used samtools (Li et al. 2009) to call SNPs and InDels. The resulting calls were filtered by coverage (≥10) and variant frequency (≥80%) to produce high-quality variant set for each of the experimental populations. We then excluded 26 variants found in experimental populations that were supported by at least two reads in the corresponding founder genotype. We also excluded two variants that were supported by at least two reads in more than one experimental population but had zero coverage in the founder genotype. There were only two variants that reached the frequency 80% in more than one independent population and were not supported by reads in the founder genotype (mutations in protein Pa_7_7970, protein site 183 in populations B2 and S5, and site 178 in populations B3 and S2). We further masked low complexity regions of founder genomes by repeatmasker (repeatmasker.org; last accessed September 2019) and excluded indels that fell into the masked sites and adjacent regions 10 bp around them. In populations A4 and B2, we observed a couple of mutations in adjacent genomic sites; we excluded these variants as probable dinucleotide mutations. According to Illumina data, none of the mutations observed in B populations at time points 1 or 2 was lost at the point 4.

### Sanger Sequencing

Sanger sequencing was used to check the presence of the called variants (138 SNPs and 24 indels) at each of the four time points. Several samples of passage 64 were lost due to *Penicillium* contamination, so we used the closest available passage 75 as the first time point passage. As each of the mutations observed at earlier time points in B populations were always present in later populations, we considered a mutation as verified if it was confirmed by Sanger sequencing in any of the four time points (assuming that the mutation that appeared once became fixed at later time points). For B populations, we believed in NGS data for passages 64 and 130 if the mutation was identified by Sanger sequencing at any time point. For A populations, we had to check each of the four time points.

Primers for Sanger sequencing were designed using primer3 (Koressaar and Remm 2007; Untergasser et al. 2012) and tntblast (Gans and Wolinsky 2008) software and synthesized in Evrogen. PCR was performed using Encyclo kit (Evrogen). Amplification products were sequenced in both directions on ABI Prism 3500 sequencer (Applied Biosystems). Chromatograms were validated manually in CodonCode Aligner (www.codoncode.com/aligner/; last accessed September 2019).

The detailed results of Sanger sequencing are included in supplementary table S2, Supplementary Material online. The number of confirmed mutations is provided in supplementary table S3, Supplementary Material online. We were unable to confirm neither the presence nor the absence of 15 mutations and excluded them from any further analyses. The majority of mutations that we failed to validate comprise mutations in noncoding/not annotated regions. In addition, there were three mutations in A populations for which we could not track the time of their appearance.

### Variant Annotation

We mapped reference CDS sequences (http://podospora.i2bc.paris-saclay.fr, v. 6.31, last accessed ∼ Dec 2014) onto the reference genome and obtained coding regions coordinates using Splign (Kapustin et al. 2008). Out of 10,635 reference protein-coding genes, we were able to identify 10,230 genes in the reference genome that coded complete proteins with proper start and stop codons. We then performed pairwise genome alignment of A and B founder genotype assemblies with published genome using Multiz (Blanchette et al. 2004) and identified coding regions coordinates in the assembled genomes using Splign annotation results. About 9,732 and 9,732 (numbers are indeed the same but protein sets differ) complete genes were identified in A and B founder genotypes, respectively, with a tiny amount of incomplete genes (627 and 530 genes for A and B populations, respectively). Based on the produced annotations, the identified genomic variants were classified as exonic, intronic, or intergenic. Exonic variants in complete genes (i.e., genes with all of their exons completely identified in the founder genome) were further classified as missense, nonsense, or silent mutations in case of SNPs and frameshift or amino acid insertion/deletion mutations in case of indels.

### Permutations

**Fig. 2.**
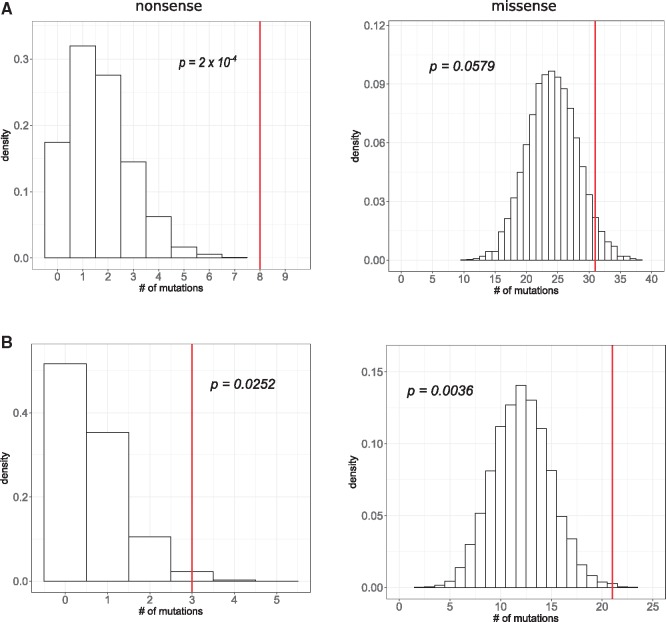
—The observed number of missense and nonsense substitutions is greater than expected by chance. Distributions of the expected number of nonsense (left panel) and missense (right panel) substitutions in A (*A*) and B (*B*) experimental populations in 10,000 permutations. Red line corresponds to the observed number of nonsense or missense substitutions in the left and the right panel, respectively. *P* (*P* value) is the area to the right of the red line.

In order to estimate the probability to obtain the observed number of protein-altering variants by chance, we generated null distributions for the number of missense and nonsense mutations in A and B founder genomes using permutations. A permutation procedure was performed as follows. About 85 and 40 observed SNP mutations were thrown at random onto A and B genomes, respectively, and annotated as described earlier. To control for possible confounding effects of mutation context, we added three-nucleotide sequence contexts to permutations. The procedure was repeated 10,000 times and produced null distributions for the expected number of nonsense and missense mutations in each of the founder genomes (fig. 2). To obtain the expected number of proteins evolving in parallel, we combined permutation results from A and B populations and counted the number of proteins that acquired at least two single-nucleotide protein-changing mutations in eight populations during the permutation procedure.

### Protein Conservation Analysis

We used OrthoMCL (Li et al. 2003) to identify orthologous groups between protein sets from *P. anserina* and *N.crassa* (Krumlau and Marzluf 1979) reference genomes. Orthologous protein pairs were then aligned using MAFFT (Katoh et al. 2002) (with –localpair option), and for each protein carrying ortholog in *N. crassa* genome, we calculated its conservation as a fraction of conserved positions in the alignment. We also measured the conservation of mutated positions as a fraction of conserved positions among the whole set of mutated positions.

## Results

### Allele Fixations Observed over the Course of the Experiment

We used two founder genotypes, denoted A and B, to establish eight replicate experimental populations (five from A and three from B, referred to as A populations and B populations). Populations were cultivated via serial passages in the standard synthetic medium M2 as described in Materials and Methods. By passage 75, five populations already transitioned to the adapted phenotype with light-colored, fluffy mycelium, and three populations (A4, A5, and B2) were still in the intermediate phase of adaptation still losing their pigmentation. By passage 130, all the populations displayed identical phenotypic changes, including changes in colony morphology and pigmentation (Kudryavtseva et al. 2011). In order to identify genetic changes acquired by the populations during the course of the experiment, we sequenced both the founder genotypes and each of the populations at four time points (fig. 1).

The summary of the observed fixations is provided in table 1 and their full list is presented in supplementary table S2, Supplementary Material online. Our set of fixations comprises 85 (73% transitions) and 40 (53% transitions) nucleotide substitutions and 13 and 10 indels in A and B populations, respectively. Among the 148 identified fixations, 52 result in an amino acid substitution, 11 are single-nucleotide substitutions leading to a premature in-frame stop codon, and 13 cause a frameshift, comprising 35.1%, 7.4%, and 8.8% of all fixed mutations, respectively.

**Table 1:** Summary of Fixations That Occurred in the Experimental Populations

Type of a Fixation	Experimental Populations A	Experimental Populations B
Total	98	50
By mutation type (single nucleotide mutations):		
Transition	62	21
Transversion	23	19
By mutation effect		
Missense nucleotide substitutions	31	21
Synonymous nucleotide substitutions	6	7
Nonsense nucleotide substitutions	8	3
Nucleotide substitutions within noncoding regions	26	5
Nucleotide substitutions within genome regions that cannot be aligned with the reference genome	9	4
Nucleotide substitutions within introns	3	0
Nucleotide substitutions in incomplete proteins	2	0
Short insertions/deletions (including frameshifts)	13 (9)	10 (4)

**Fig. 3.**
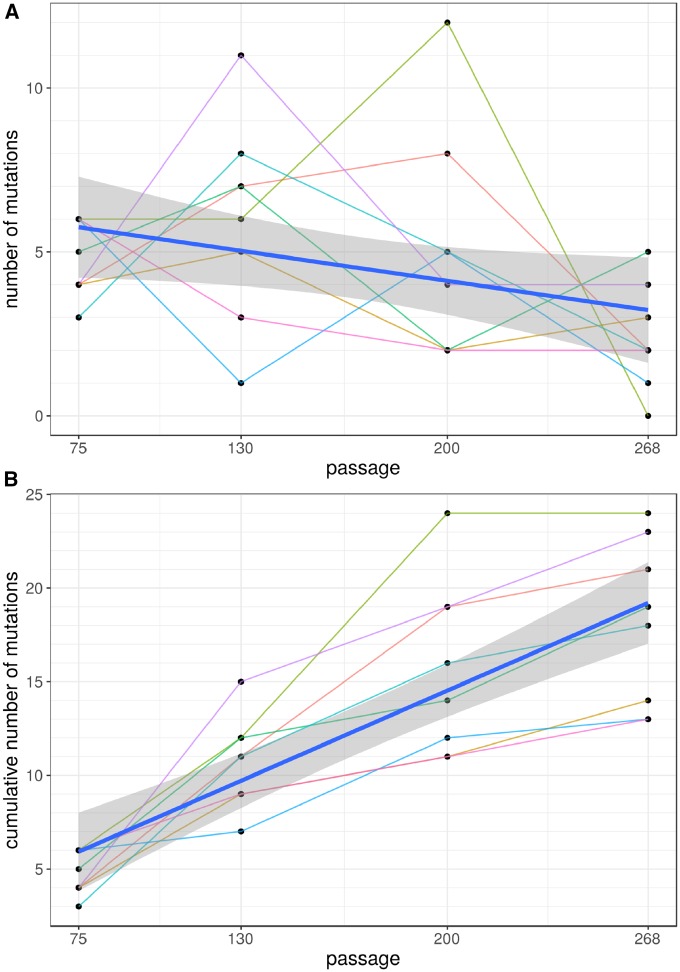
—Accumulation of fixations during the experiment. Each dot reflects the number of fixations observed in a population at a particular time point (*A*) or the cumulative number of fixations (*B*). Populations are shown as thin lines. Thick blue line corresponds to the linear regression line with confidence interval depicted as shaded area.

The dynamics of accumulation of fixations is summarized in figure 3 and supplementary table S3, Supplementary Material online. We observed a slight decrease in their rate over the course of the experiment (fig. 3*A*, the estimate for the linear regression’s time variable coefficient is −0.013), but this decrease was only marginally significant (*P* value = 0.0491). On an average, 14 passages are required for one fixation to take place (fig. 3*B*, see supplementary table S3, Supplementary Material online). There were no mutators: the numbers of mutations in each population were indistinguishable and there was no difference between A and B populations (two-way ANOVA test, see supplementary table S3, Supplementary Material online).

Besides fixations, we observed a substantial number of low-to-intermediate frequency variants (supplementary fig. S1, Supplementary Material online), although some of these may arise artifactually due to noise in the NGS data and low sequencing coverage in our experiment.

### Distribution of Fixations within the Genome

We then focused on the analysis of single nucleotide substitutions and first computed the d*N*/d*S* ratio, which equaled 1.48 (supplementary table S4, Supplementary Material online). While d*N*/d*S* >1 is commonly interpreted as indication of positive selection, our estimates may be unreliable because the numbers of both nonsynonymous and synonymous mutations were low (binomial test *P* value = 0.22; see supplementary table S4, Supplementary Material online). To investigate how the fixed mutations are distributed in the *P. anserina* genome, we first produced null-distributions for the number of missense and nonsense substitutions using a permutation procedure (see Materials and Methods). Null-distributions were obtained separately for A and B populations, assuming that mutations hit the genome and become fixed at random, with uniform per site rates. There are significantly more nonsense substitutions than expected by chance in both A and B populations, and significantly more missense substitutions in B populations (fig. 2).

**Fig. 4.**
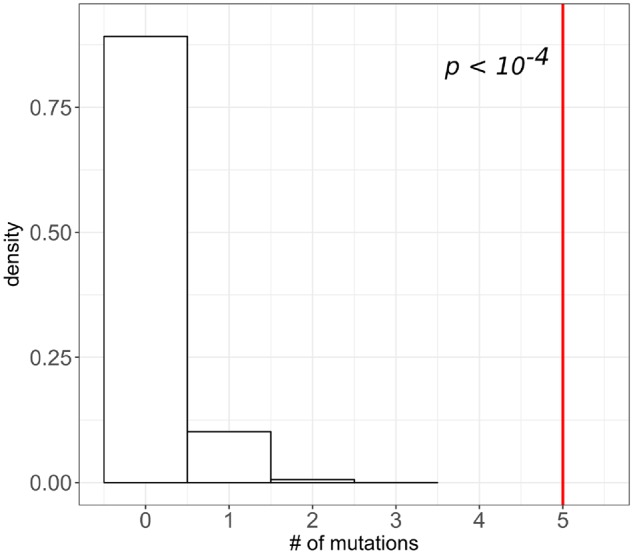
—The excess of the number of proteins with parallel changes. Distribution of the number of genes with two protein-altering single-nucleotide substitutions in 10,000 random permutations. Red line shows the observed number of genes with two or more such substitutions occurred.

Moreover, despite the fact that there were very few mutations overall, a substantial proportion of fixed protein-altering mutations affected the same gene and protein in more than one population. Totally, six proteins were subject to such parallel evolution (table 2). The probability of observing the same or a higher number of genes carrying just two single-nucleotide protein-altering substitutions by chance is very low (fig. 4), and four out of six proteins that underwent parallel evolution accepted more than two fixations. Clearly, positive natural selection was responsible for this phenomenon.

**Table 2:** Proteins Affected Independently in More Than One Population

Protein Identifier and Its Function	The Number of Independent Fixations
Pa_1_10140, Putative transcriptional regulatory protein pro-1	2 nonsense substitutions
Pa_1_23950, Guanine nucleotide-binding protein alpha subunit encoded by the *mod-D* gene	1 nonsense substitutions, 2 missense mutations
Pa_3_400, putative protein with unknown function	6 frameshifts
Pa_3_6550, Putative protein homologous to VeA of *Aspergillus nidulans*	2 missense substitutions
Pa_6_3340, Putative developmental regulator flbA	2 nonsense substitutions, 1 missense substitutions
Pa_7_7970, Putative guanine nucleotide-binding protein alpha-1 subunit	7 missense substitutions

This parallelism cannot be an artifact of ancestral polymorphism segregating within our lineages, since we conservatively excluded a number of positions with identical parallel mutations in multiple populations founded from the same ancestral lineage. For the same reason, we could lose some actual functional parallelism, making our estimates for the frequency of parallel substitutions conservative. We did observe two identical nucleotide mutations between two lineages formed from two different ancestral lineages (supplementary table S2, Supplementary Material online); those are unlikely to be due to ancestral polymorphism.

### Proteins Carrying Missense and Nonsense Substitutions Are More Conservative

To study the rate of long-term evolution of proteins that evolved in our experiment, we searched for pairs of orthologs pairs encoded by *P. anserina* and *N.crassa* genomes. Out of 10,230 proteins in the reference *P. anserina* genome, 4,713 have an ortholog in *N. crassa* genome, such that their alignments contain no more than 20% of gaps. Both nonsense and, to a lesser extent, missense fixations preferentially occurred in those *P. anserina* proteins that have reliable orthologs in *N. crassa* (table 3).

**Table 3:** Statistics of Orthologization of *Podospora anserina* and *Neurospora crassa* Genomes

	Total No. of Proteins	No. of Proteins Carrying Nonsense Substitutions	No. of Proteins Carrying Missense Substitutions
No. of proteins in *P. anserina* genome	10,230	9	44
No. of such proteins that have a reliable ortholog in *N. crassa*	4,713	9 (0.000935)	27 (0.04888)

Note.—Values specified in parentheses correspond to *P* values of binomial test.

Moreover, missense fixations within such proteins preferentially occurred at amino acid sites conserved between the two species. While the overall proportion of such sites for 4,713 alignments of reliable orthologs is 62%, 87% (27 out of 31) of replaced amino acids occupied conservative sites (*P* = 0.003).

### Possible Phenotypic Effects of Parallel Fixations

Proteins that underwent fixations in more than one experimental population (table 2) are of particular interest. Indeed, their evolution must have been adaptive (fig. 4) and, therefore, must have led to changes in the phenotype. Moreover, they probably affected the protein structures radically. Indeed, the differences in physicochemical properties (computed as Miyata’s distance) for pairs of amino acids were higher for missense substitutions that occurred during our experiment than for those that occurred in the course of divergence between *P. anserina* and *N. crassa* (mean values 1.979811 vs. 1.472333, two-tail *t*-test *P* value 0.002).

Let us discuss what these changes could be.

The most striking example of parallel evolution is protein Pa_7_7970 which experienced nucleotide substitutions in seven independent populations out of 8. Pa_7_7970 is a putative alpha-1 subunit of a guanine nucleotide-binding protein (table 2). There are three classes of G protein alpha subunits in ascomycetes fungi (Bölker 1998), each affecting fungal growth and development differently. Pa_7_7970 belongs to class 1 and is 93% identical to its well-studied orthologous protein FadA of *Aspergillus nidulans*. FadA stimulates vegetative growth (Krijgsheld et al. 2013) and probably suppresses sexual reproduction (Han et al. 2008), which agrees well with intensive vegetative growth during the experiment suggesting that constitutive activation of Pa_7_7970 may be adaptive under the conditions of submerged cultivation.

Only four sites in Pa_7_7970 evolved in the course of the experiment: arg-178 was substituted in three experimental populations, gly-183 was substituted twice, and ser-206 and lys-210 were each substituted once. All these sites have already been studied. Arg-178 is a catalytic residue crucial for GTP hydrolysis and alpha subunit inactivation (Coleman et al. 1994) and its mutations result in constitutive activation of alpha subunit (Landis et al. 1989). Gly-183 takes part in the interaction between alpha subunit and regulator of G protein signaling (RGS) that activates GTP hydrolysis, and Gly->Ser substitution (observed in one case) hinders this process drastically (Lan et al. 1998). Ser-206 and Lys-210 are also involved in the interaction with RGS (Clark and Lambert 2006; Khafizov et al. 2009) although no specific effects of their mutations have yet been described.

Remarkably, we also observed one missense and two nonsense substitutions in the ortholog of RGS (Pa_6_3340) which belongs to the same pathway as FadA and likely contributes to *P. anserina* adaptation in similar fashion.

Another protein that is worth mentioning is a transcription factor pro-1 (Pa_1_10140) which acquired two loss-of-function fixations. pro-1 interacts with promoters of many developmental genes including *esdC* gene that stimulates sexual development and is suppressed by FadA (Clark and Lambert 2006; Khafizov et al. 2009), and deletion of *pro-1* results in sexual sterility. Since *P. anserina* proliferates only vegetatively during the experiment, defects in sexual development pathway may be beneficial.

We observed two missense substitutions in VeA homolog (Pa_3_6550). Although we cannot suggest the effect of two missense substitutions on protein function, VeA in *A.nidulans* positively regulates expression of the previously mentioned *esdC* gene (Han et al. 2008) and might affect *P. anserina* metabolism in some way.

We also observed one protein with a surprising amount of parallel evolution via frameshifts. Pa_3_400 protein experienced frameshift in six independent populations. Its homologs in *N.crassa* (Springer and Yanofsky 1989) and *A. nidulans* are involved in asexual reproduction (Chung et al. 2011). As *P. anserina* lacks asexual reproduction, we cannot suggest the role of Pa_3_400 inactivation in *P. anserina* adaptation, although in *P. anserina* it can be involved in other developmental pathways.

As for Pa_1_23950, it belongs to class 3 alpha subunits of fungal G proteins. Mutations in this protein cause defects in vegetative proliferation in *P. anserina* (Loubradou et al. 1999) making it difficult to interpret the observed substitutions in this protein in the context of adaptation.

All proteins discussed above are involved in growth and development processes in various fungi. Taken with the excess of coding substitutions and the number of protein changed in parallel, it favors the possible adaptiveness of some of the observed fixations toward the conditions of submerged cultivation. Yet mutant construction is needed to claim it with certainty.

## Discussion

Submerged cultivation is an unusual and poorly explored system in the context of experimental evolution. One work on submerged cultivation used ascomycetous fungus *Trichoderma citrinoviride* that evolved hyphal fragmentation, efficient conidia production, and faster germination (Lin et al. 2017). The acquired physiological changes, including spore production atypical under submerged cultivation, allowed the fungus to transfer effectively between passages via liquid aliquots. Interestingly, *P. anserina* cultures in our experiment were transferred between passages as mycelial fragments but still evolved more dispersed mycelium compared with unadapted ancestral genotype.

In populations of *P. anserina*, transition to prolonged submerged cultivation inevitably results in the same set of morphophysiological changes (Turker and Cummings 1987). These changes always occur early in the course of an experiment (Kudryavtseva et al. 2011) and probably can be mostly explained by modifications of epigenetic patterns that alter gene expression. Yet at a longer timescale, one can also expect *P. anserina* to adapt to the conditions that are far from its native environment through fixations of newly acquired mutations. To our knowledge, this is the first work that studies the genetic aspects of adaptation of a complex fungal organism to prolonged submerged cultivation.

We analyzed small-scale changes that occurred in the *P. anserina* genome in the course of 268 experimental passages. We observed and confirmed the appearance of 125 single-nucleotide substitutions and 23 short indels. It is hard to tell which of these mutations are advantageous for submerged cultivation. Experimental populations of *P. anserina* cannot reproduce sexually under conditions of the experiment, and *P. anserina* genus itself lacks the ability to form asexual reproductive structures, macroconidia. Thus, the propagation of our experimental populations occurred exclusively through vegetative proliferation, so that they must possess clonal population structure. Thus, hitch-hiking of neutral and even slightly deleterious mutations that arose in high-fitness genotypes destined to fixation was likely common (Barton 2000). Nevertheless, two observations clearly indicate that positive selection drove many of the observed fixations which, therefore, contributed to adaptation to the experimental environment.

First, evolution of our eight experimental populations often occurred in parallel, with the same gene and protein being affected by a mutation up to seven times (table 2). Parallel evolution of phenotypes is common in nature, although it does not necessarily involve parallel genetic changes (Steiner et al. 2009). In contrast, in evolutionary experiments parallel genetic changes have being reported several times (Wichman et al. 1999; Woods et al. 2006; Lang et al. 2013; Lang and Desai 2014). Parallelisms at nucleotide level are rare, and we observed only two such events in Pa_7_7970 gene, which might be mutational hotspots but considering the importance of the mutated protein sites clearly indicate their adaptive role.

Some of the six proteins that underwent parallel changes in our experiment are involved in the same pathways, and all of them seem to be associated with growth and development processes in fungi. A prominent role of nonsense and frameshift alleles, as well as the nature of the fixed missense alleles, indicates that this parallel evolution mostly, if not exclusively, led to inactivation of the affected proteins. At least five missense substitutions in Pa_7_7970, as well as nonsense substitutions in Pa_6_3340, alter developmental pathways probably stimulating vegetative growth and inhibiting sexual reproduction at a very early stage. A similar pattern was observed in experimental yeast populations, where parallel evolution affected genes of the mating pathway and small GTPases of the Ras protein family (Lang et al. 2013). Loss-of-function mutations of large effects can provide a selective advantage inactivating parts of regulatory/metabolic network that became useless under the current environmental conditions (Hottes et al. 2013). For example, in yeasts, mutations resulting in sexual sterility provide advantage in growth rate (Lang et al. 2009).

Still, the extent of parallel evolution in our experiment is remarkable. Fungi tend to not differentiate in liquid medium (Osiewacz 2002), so that sexual reproduction was already suppressed before experimental evolution ever begun. Thus, here, the situation is very different from parallel loss of eyes in many cave populations of *Astyanax mexicana*, where the cost of maintaining even a useless capacity to see was estimated as up to 15% of resting metabolism (Moran et al. 2015), which makes its elimination obviously beneficial. In contrast, we observed that positive selection strongly favored mutations that damage a top-level metabolic pathway which already lacks a salient phenotypic expression. Perhaps, before loss-of-function mutations in regulatory proteins occur, some cryptic activity of this pathway remains, and fixation of such mutations allows cells to reallocate their resources to vegetative proliferation. Thus, the role of positive selection in degenerative evolution may be even more prominent than thought previously.

The second reason to believe that many of the observed fixations were adaptive is overrepresentation among them of nonsense and missense mutations which must have disproportionally strong impact on function (fig. 2). Moreover, among missense mutations, those that involve radical amino acid replacements and those that occurred at conservative sites and in more conservative proteins are also more common than expected. Because such mutations have a reduced chance of being effectively neutral, this pattern implies that positive selection drove most of their fixations.

Surprisingly, there is still no clarity regarding the role of functionally radical mutations in the adaptive evolution in nature. The radical-conservative ratio of missense substitutions has been studied in the context of both positive and negative selection, and while there are some hints that it correlates with the strength of negative selection (Smith 2003; Popadin et al. 2007; Wernegreen 2011), its relation to positive selection remains ambiguous. Radical-conservative ratio has been proposed as a measure to detect positive selection almost three decades ago (Hughes et al. 1990). However, this measure is vulnerable to various factors not related to selection (Dagan et al. 2002) and studies of different species did not find enough evidences for the excess of radical compared with conservative substitutions at positively selected sites (Smith 2003; Hanada et al. 2006; Suzuki 2007).

A prominent role of mutations at conservative sites and proteins in adaptation to the experimental environment was reported for *E. coli* (Maddamsetti 2017). An obvious possibility is that these, as well as ours, data reflect how adaptation occurs in nature. Indeed, substitutions at conservative sites that occur in the course of natural evolution are enriched with those driven by positive selection (Bazykin and Kondrashov 2012). Alternatively, a disproportional contribution of radical mutations to experimental evolution may also be due to simplified laboratory environments, under which adaptation mostly involves shedding functions that are no longer needed. Clearly, this issue deserves to be studied further.

In the course of the experiment, our populations may experience not only adaptation toward submerged cultivation but also other processes typical for such systems, such as clonal interference and ecological coexistence. Unfortunately, in contrast to detection of adaptation, the signal of interaction between different clones is difficult to unravel. During the experiment, we observed a number of variants at low-to-intermediate frequency supplementary fig. S1, Supplementary Material online). Although some of them may be errors introduced by the NGS or variant calling procedure, others can result from the action of genetic drift, natural selection, or interaction between clones. The analysis and interpretation of the dynamics of these variants is hindered by the lack of information on mutation phasing, a limited number of time points and unknown effective population size.

At the moment, there is no obvious trend in mutation accumulation rate. Experimental populations acquired two times less mutations between time points 3 and 4 than they did in the previous time interval. However, the reason for that is yet unclear, and further observations might shed some light on this tendency. The experiment lasts for 6 years already, and to our knowledge, there are few long-lasting evolutionary experiments conducted on multicellular organisms. We are planning to continue the experiment and analyze changes in experimental populations of *P. anserina*. 

## Supplementary Material

Supplementary data are available at *Genome Biology and Evolution* online.

## Supplementary Material

evz194_Supplementary_Data

## Notes

**Data Deposition:** Genome sequence data have been deposited at DDBJ/ENA/GenBank under accessions QHKV00000000 (founder genotype A; version QHKV01000000) and QHKU00000000 (founder genotype B; version QHKU01000000), with the respective BioSample accessions SAMN09270751 and SAMN09270757, under BioProject PRJNA473312. Sequencing data have been deposited at the SRA with accession numbers SRR7233712–SRR7233727, under the same BioProject.
