# Supplementary material for: Genetics of Adaptation of the Ascomycetous Fungus Podospora anserina to Submerged Cultivation
Source: Genome Biol Evol. 2019 Sep 14;11(10):2807–17. doi: 10.1093/gbe/evz194 (PMC6786475; doi:10.1093/gbe/evz194)
Supplement: evz194_Supplementary_Data [file evz194_supplementary_data.zip › Figure_S1.pdf]

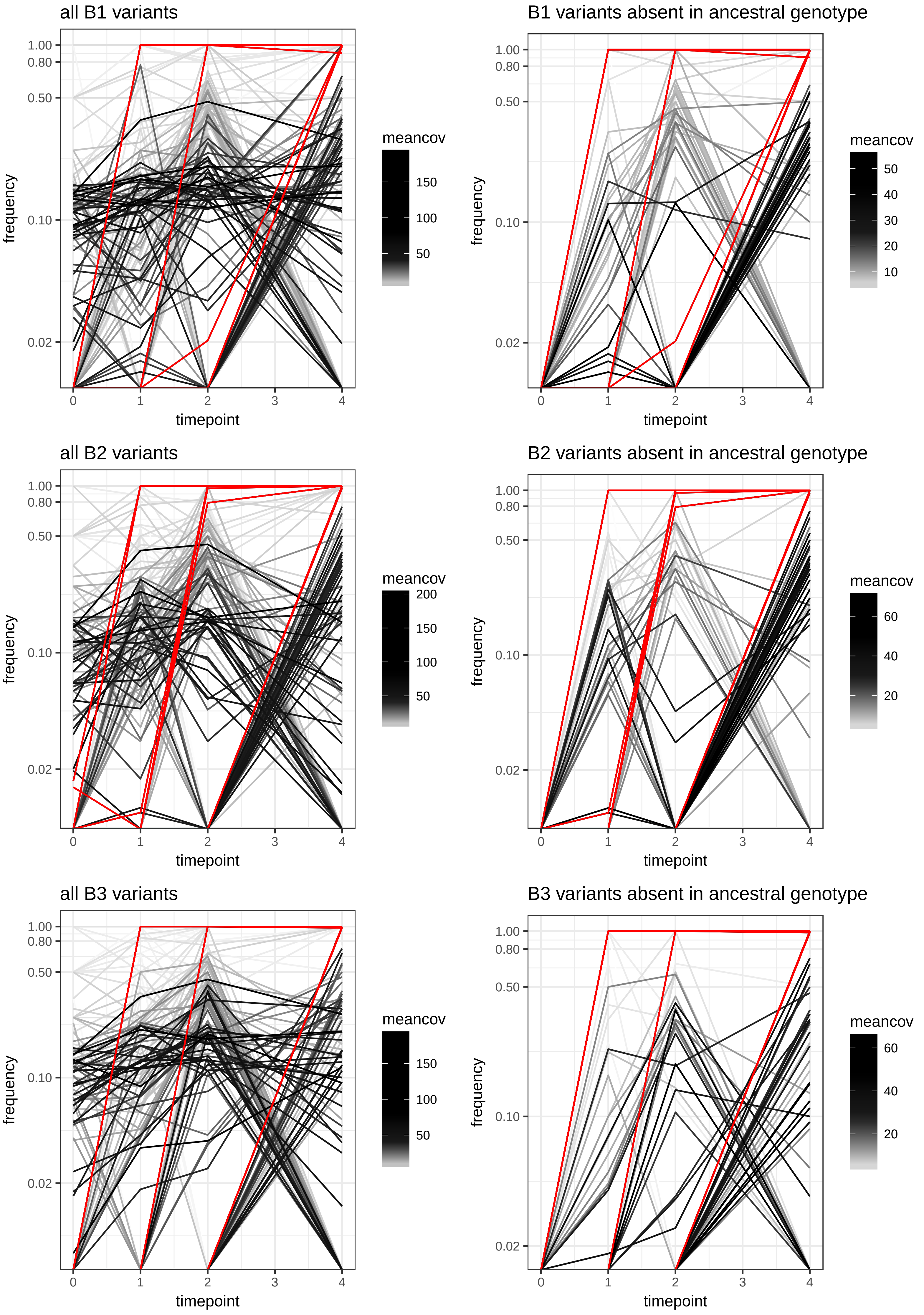
all B1 variants
frequency
1.00
0.80
0.50
0.10
0.02
timepoint
0 1 2 3 4
meancov
150
100
50
B1 variants absent in ancestral genotype
frequency
1.00
0.80
0.50
0.10
0.02
timepoint
0 1 2 3 4
meancov
50
40
30
20
10
all B2 variants
frequency
1.00
0.80
0.50
0.10
0.02
timepoint
0 1 2 3 4
meancov
200
150
100
50
B2 variants absent in ancestral genotype
frequency
1.00
0.80
0.50
0.10
0.02
timepoint
0 1 2 3 4
meancov
60
40
20
all B3 variants
frequency
1.00
0.80
0.50
0.10
0.02
timepoint
0 1 2 3 4
meancov
150
100
50
B3 variants absent in ancestral genotype
frequency
1.00
0.80
0.50
0.10
0.02
timepoint
0 1 2 3 4
meancov
60
40
20
