# Supplementary material for: Genetics of Adaptation of the Ascomycetous Fungus Podospora anserina to Submerged Cultivation
Source: Genome Biol Evol. 2019 Sep 14;11(10):2807–17. doi: 10.1093/gbe/evz194 (PMC6786475; doi:10.1093/gbe/evz194)
Supplement: evz194_Supplementary_Data [file evz194_supplementary_data.zip › Figure_S2.pdf]

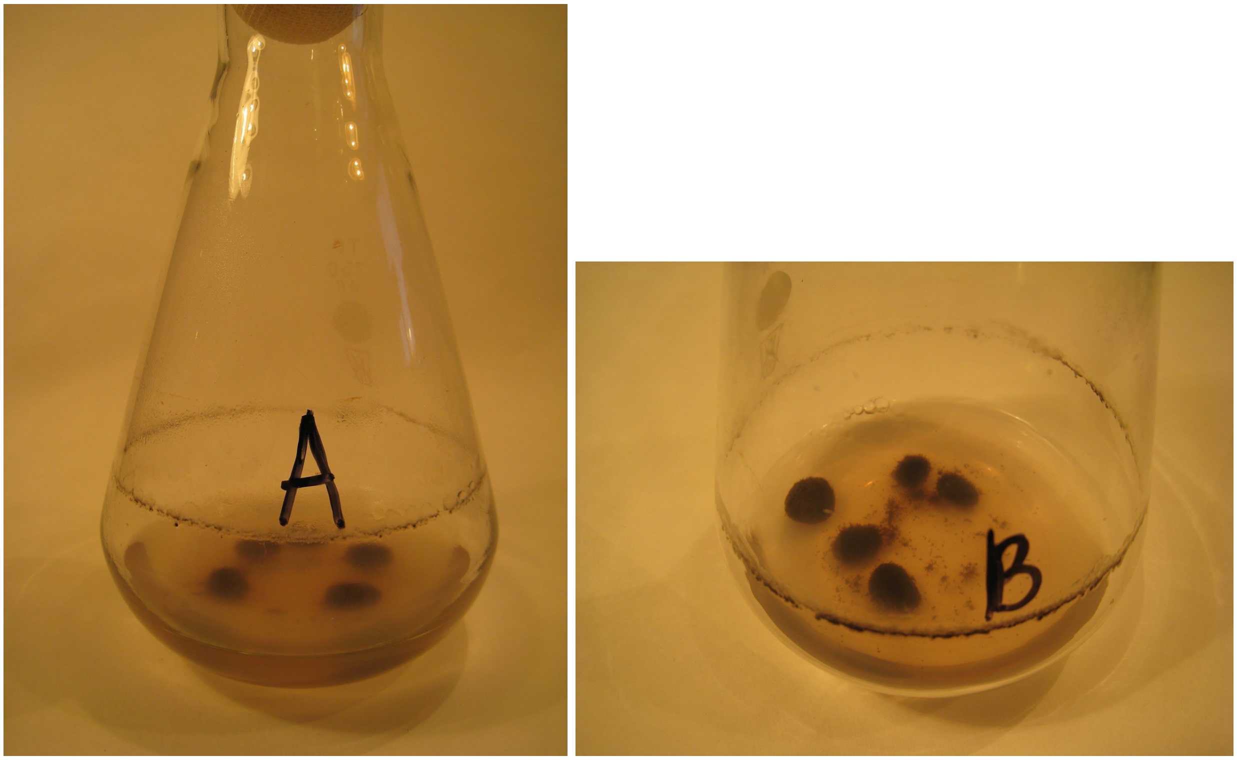

I. Ancestral genotypes A and B (passage 0) demonstrate dark pellets after submerged inoculation.

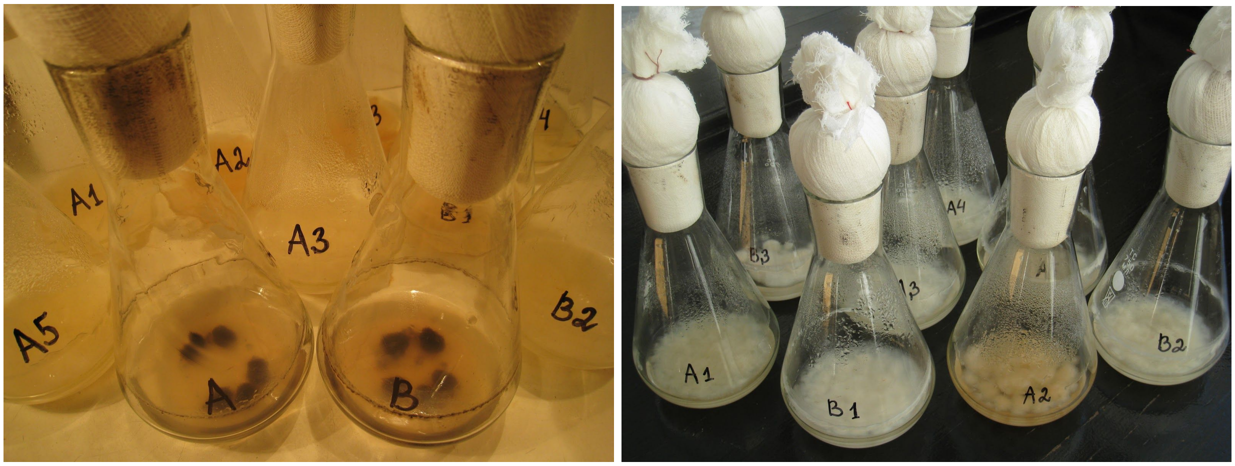

II. Adapted experimental populations A1-A5 and B1-B3 (passage 460) acquired a light, more dispersed mycelium. Left, experimental populations changed their pigmentation and mycelium morphology compared to ancestral genotypes A and B. Right, experimental populations are placed against a dark background. Population A2 has been demonstrating red pigmentation since the first time point (passage 75).
