# Supplementary material for: Genetics of Adaptation of the Ascomycetous Fungus Podospora anserina to Submerged Cultivation
Source: Genome Biol Evol. 2019 Sep 14;11(10):2807–17. doi: 10.1093/gbe/evz194 (PMC6786475; doi:10.1093/gbe/evz194)
Supplement: evz194_Supplementary_Data [file evz194_supplementary_data.zip › Suppl legends.docx]

# Supplementary Information

**Table S1. Statistics on founder genotypes assemblies and average mapping coverage.**

**Table S2. The summary of the observed fixations.** Sheets A1-A5 and B1-B3 contain the results of the Sanger sequencing validation procedure. The two last sheets comprise annotation of the fixations in A and B populations.

**Table S3. Accumulation of fixations.** Each entry provides the number of newly observed fixations, relatively to the previous time point.

**Table S4. dN/dS calculation.**

**Figure S1. Frequency dynamics of the observed single nucleotide variants.** For this analysis, we excluded genome positions that were not covered by at least one of time points; we next excluded variants that were located less than 100bp away from each other in the same time point. Traces of the fixed variants are shown in red. On the right panel, variants that were supported by at least one read in the ancestral genotype were discarded. Note that this strict filter removed two confirmed variants in B2.

**Figure S2. Photographs of the ancestral genotypes and experimental populations.**
